# Pubertal timing and breast cancer risk in the Sister Study cohort

**DOI:** 10.1186/s13058-020-01326-2

**Published:** 2020-10-27

**Authors:** Mandy Goldberg, Aimee A. D’Aloisio, Katie M. O’Brien, Shanshan Zhao, Dale P. Sandler

**Affiliations:** 1 Epidemiology Branch, National Institute of Environmental Health Sciences, 111 TW Alexander Dr, Research Triangle Park, NC 27709 USA; 2 Social and Scientific Systems, Durham, NC USA; 3 Biostatistics & Computational Biology Branch, National Institute of Environmental Health Sciences, Research Triangle Park, NC USA

**Keywords:** Breast cancer, Puberty, Menarche, Breast development, Thelarche

## Abstract

**Background:**

Earlier age at menarche is an established risk factor for breast cancer. While age at menarche has been fairly stable over the past half-century, age at breast development (thelarche) has continued to decrease. Recently, earlier age at thelarche and a longer time between thelarche and menarche (pubertal tempo) were shown to be associated with increased breast cancer risk. Our objective was to examine how breast cancer risk was associated with pubertal timing and tempo in a prospective US cohort.

**Methods:**

Women ages 35–74 years without a history of breast cancer, but who had a sister previously diagnosed with breast cancer, were enrolled in the Sister Study from 2003 to 2009 (*N* = 50,884). At enrollment, participants reported their ages at thelarche and menarche. Pubertal tempo was age at menarche minus age at thelarche. We estimated adjusted hazard ratios (HRs) and 95% confidence intervals (CIs) for each pubertal milestone and risk of breast cancer (invasive or ductal carcinoma in situ) using Cox proportional hazards regression. We examined whether associations between age at thelarche and breast cancer risk were modified by birth cohort, race/ethnicity, weight at age 10, and extent of breast cancer family history, as characterized by a Bayesian score based on first-degree family structure.

**Results:**

During follow-up (mean = 9.3 years), 3295 eligible women were diagnosed with breast cancer. Early ages at thelarche (HR = 1.23, 95% CI 1.03–1.46 for < 10 vs. 12–13 years) and menarche (HR = 1.10, 95% CI 1.01–1.20 for < 12 vs. 12–13 years) were positively associated with breast cancer risk. Pubertal tempo was not associated with breast cancer risk (HR = 0.99, 95% CI 0.97–1.02 per 1-year longer tempo). When considering early thelarche (< 10 years) and early menarche (< 12 years) jointly, women with both had a 30% greater risk of breast cancer compared with women with neither risk factor (95% CI 1.07–1.57). The association between age at thelarche and breast cancer risk did not significantly vary by birth cohort, race/ethnicity, childhood weight, or Bayesian family history score.

**Conclusions:**

Earlier ages at thelarche and menarche may enhance susceptibility to breast carcinogenesis. Age at thelarche is an important risk factor to consider given secular trends towards earlier development.

## Background

Earlier age at menarche is an established risk factor for breast cancer [1]. Age at menarche has historically decreased over time, but has remained fairly stable over the past half-century [2]. In contrast, age at onset of breast development (thelarche), which occurs on average 2–3 years before menarche [3], has continued to decline rapidly [2, 4–6]. Causes of this rapid decline in age at thelarche are not known, but increasing rates of childhood obesity and changes in environmental factors, such as exposure to endocrine-disrupting chemicals and psychosocial stressors, are hypothesized to contribute to this trend [2, 6, 7]. Puberty is a period of rapid breast development and a window of susceptibility for breast cancer risk, as the developing tissue may be particularly vulnerable to carcinogenesis [8]. Markers of pubertal development, such as thelarche and menarche, are associated with hormonal changes and breast growth [9] and are used to estimate the duration of this vulnerable period.

 In contrast to age at menarche, few studies have examined other pubertal markers in relation to breast cancer risk, particularly age at thelarche. The timing of thelarche may be more biologically relevant to breast cancer risk than the timing of menarche as thelarche represents the onset of the vulnerable window of rapid breast development*.* Earlier thelarche and longer time period between thelarche and menarche were independently associated with a 20–30% increased risk of breast cancer in the Generations Study cohort [10]. In that study, earlier age at reaching adult height was also associated with increased breast cancer risk [10]. Age reached adult height is correlated with age at peak height velocity [11], a pubertal marker which occurs on average 1 year before menarche [12]. An inverse relationship between age reached adult height and breast cancer risk has also been observed in other [13–17], but not all [18], studies.

Our objective was to examine associations between pubertal markers and breast cancer risk in a prospective US cohort. These markers include age at thelarche, age at menarche, age at attainment of adult height, and the timing between these events. Given recent secular declines in age at thelarche [2, 4, 5], we further evaluated whether factors associated with early thelarche, including race/ethnicity [4, 19], childhood weight [20], and extent of breast cancer family history [21], modified associations between age at thelarche and breast cancer risk.

## Methods

### Study population

The Sister Study is a prospective cohort designed to investigate environmental and genetic risk factors for breast cancer (for more details, see [22]). From 2003 to 2009, 50,884 women enrolled in the cohort. Women were eligible if they lived in the USA including Puerto Rico, were between the ages of 35 and 74 years, and did not have a personal history of breast cancer, but had a sister previously diagnosed with breast cancer.

Women completed a computer-assisted telephone interview at baseline which included information on demographics, medical and family history, and reproductive and lifestyle factors. During follow-up, women are asked to complete annual health updates and more extensive questionnaires every 2–3 years. Response rates have ranged from 91 to 96% throughout follow-up [22]. This analysis utilized Sister Study Data Release 7.2, including follow-up data up to September 15, 2017.

All participants provided written informed consent. The institutional review boards of the National Institute of Environmental Health Sciences, National Institutes of Health, and the Copernicus Group approved the study.

### Incident breast cancer

Participants reported diagnoses of breast cancer on annual health updates and detailed follow-up questionnaires. Women who reported an incident breast cancer were asked for diagnostic information and permission to obtain medical records, including pathology reports. Medical records and/or pathology reports were obtained for more than 80% of women with a self-reported breast cancer. Agreement between self-reports and medical records has been high (positive predictive value > 99%) [23], so self-reports were used when medical records or pathology reports were not available. We examined incident breast cancer (invasive breast cancer or ductal carcinoma in situ (DCIS)) as the primary outcome of interest.

### Pubertal timing assessment

During the baseline interview, women reported the age in years when they first noticed their breasts developing. Women who did not know the age were asked to report their grade in school, which we converted to age (1.2% of the cohort reported grade). Women also reported the age in years and months when they had their first menstrual period. If age was unknown, women were asked to report their school grade (0.3%, converted to age) or if their period started before, around the same time as, or after other girls (0.2%, imputed as 11, 12.5, and 14 years). We truncated age at menarche at years since few women reported months (~ 3%). Women responded to the question “How old were you when you first reached your full adult height? This is usually before the age of 20” with age in years. Women who did not know the age were given categorical options of 10–13, 14–17, and 18–20 years (3.2%, imputed as 12, 16, and 19 years). We calculated thelarche-menarche tempo as age at menarche minus age at thelarche and menarche-adult height tempo as age reached adult height minus age at menarche.

### Analytic sample

Of the 50,884 women enrolled in the cohort, we excluded 3 women who withdrew their data from the study; 75 women diagnosed with breast cancer prior to, at the same age as, or unknown relative to the completion of all baseline study components; and 3 women with uncertain cancer diagnoses (Additional File 1: Fig. S1). We also excluded women with unknown or missing ages at pubertal milestones (*n* = 738); women with implausibly late ages (*n* = 260), defined as ≥ 21 for thelarche, ≥ 22 for menarche, and ≥ 26 for age reached adult height; and women who reported never having a menstrual period (*n* = 7). We selected these cut-offs to define implausible ages after exploring the distributions of self-reported ages at pubertal events in our data, as well as reviewing previous literature [15, 24] and considering the expected biological sequence of pubertal events. We excluded women with missing data on race/ethnicity or relative childhood family income (*n* = 112) for a final analytic sample of 49,686 women.

### Statistical analysis

We examined the distributions of early-life characteristics and pubertal variables by age at thelarche. We used Spearman correlation coefficients to examine the relationships between continuous pubertal timing and tempo variables.

We used Cox proportional hazards regression with age as the time scale to estimate hazard ratios (HRs) and 95% confidence intervals (CIs) for associations between pubertal exposures and breast cancer risk. Women accrued person-time from age at study enrollment until age at diagnosis of breast cancer or age at last study follow-up, loss to follow-up, or death. We tested for violations of the proportional hazard assumption using Wald or joint Wald tests of interaction terms between exposures and follow-up time. The assumption was not violated for the exposures of interest except where noted in table footnotes for secondary analyses.

We examined pubertal timing and tempo variables continuously and in categories. We considered models using the same categories examined in the Generations Study [10]. We stratified models by birth cohort in approximately 10-year intervals to control for potential cohort effects. We then adjusted for race/ethnicity (non-Hispanic white, non-Hispanic black, Hispanic, and others) and family income level growing up (well-off, middle income, low income, and poor), as reported by the participant at enrollment.

We modeled each pubertal exposure separately since exposures were correlated (Additional File 2: Table S1). We also present a mutually adjusted model including ages at thelarche and menarche and a model for age reached adult height adjusted for age at menarche. We present thelarche-menarche tempo models adjusted for age at thelarche and menarche-height tempo models adjusted for age at menarche to consider confounding by age at the first milestone. To further explore whether ages at thelarche and menarche were independent risk factors for breast cancer, we considered early thelarche (< 10 years) and early menarche (< 12 years) jointly using a four-category variable. We tested for interaction between early thelarche and early menarche through the Wald test of the cross-product term in a model that also included both main effects.

We examined whether associations between age at thelarche and breast cancer risk were modified by birth cohort, race/ethnicity, weight relative to peers at age 10 years (heavier vs. same weight or lighter), and height relative to peers at age 10 years (taller vs. same height or shorter) through stratification and tested for statistical heterogeneity using the Wald or joint Wald test. We excluded women with missing data for the modifier of interest in stratified models and also in models including main effects and interaction terms that tested for statistical heterogeneity (*n* = 64 women with missing childhood height and *n* = 124 missing childhood weight). We also examined effect modification by familial risk using a continuous Bayesian family history score (BFHS) incorporating the family size, number of breast cancer cases in first-degree relatives, age at diagnosis for cases, and current age or age at death for non-cases (for more details, see [25, 26]). This score was developed in the Sister Study cohort. It ranges from 0 to 1 and can be interpreted as the family-specific pure lifetime breast cancer risk [25]. We stratified by BFHS, dichotomized at the median. We excluded women who reported at enrollment that they were adopted from the BFHS analyses (*n* = 183).

We considered heterogeneity in the association between age at thelarche and breast cancer risk by tumor invasiveness, estrogen receptor (ER) status, and menopausal status at diagnosis. For models examining invasive and DCIS disease separately, women with the alternate type were censored at age at diagnosis. For ER status, we included invasive and DCIS cases and censored women with the alternative subtype, with missing subtype information, or with a borderline test result at age at diagnosis. We calculated effect estimates and tested for statistical heterogeneity by invasiveness and ER status using fully adjusted joint Cox models additionally stratified by type [27]. We censored women who transitioned from pre-menopausal to post-menopausal during follow-up at age at menopause in models of pre-menopausal cancer. In models of post-menopausal cancer, women accrued person-time from age at cohort enrollment or age at menopause, whichever occurred later. We tested for heterogeneity by menopausal status using the Wald or joint Wald test.

We conducted sensitivity analyses to consider how error in the recall of age at thelarche may have biased associations between age at thelarche and breast cancer risk. Because thelarche is expected to occur prior to menarche, we assumed that thelarche occurred either 1 year or 2 years earlier than what they had reported for women who reported that thelarche occurred at the same age or later than menarche (tempo ≤ 0 years) in order to quantify the bias arising from misclassification of age at thelarche. We also ran analyses restricting the study population to women more likely to have accurate recall. We limited models to women younger than 60 years at enrollment, as older women may be less likely to accurately report age at thelarche. In separate models, we excluded women with implausible thelarche-menarche tempo, as extreme tempos may reflect inaccurate reporting of age at thelarche. We defined implausible thelarche-menarche tempo in three ways: (1) tempo below the observed 5th percentile (menarche reported > 2 years before thelarche) or above the 95th percentile (menarche reported > 3 years after thelarche), (2) tempo < 0 years (menarche reported before thelarche) or > 4 years, and (3) tempo ≤ 0 years (menarche reported at the same age or prior to thelarche).

We recalculated thelarche-menarche tempo after adjusting age at thelarche as described above in sensitivity analyses examining thelarche-menarche tempo and breast cancer risk. We also ran tempo analyses excluding women ages 60 and older at enrollment. In addition, we conducted a probabilistic bias analysis of the thelarche-menarche tempo and breast cancer relationship. To do so, we first evaluated the distribution of pubertal tempo among women who reported that menarche occurred at least 1 year after thelarche. We then assigned women with a thelarche-menarche tempo ≤ 0 years a new tempo value based on a random draw from that distribution. We ran this simulation 20 times and combined the measures of association from fully adjusted Cox models across datasets using Rubin’s rules [28].

We used robust variance estimates to account for correlations between sisters in the study sample. We conducted analyses using SAS 9.4 (SAS Institute Inc.).

## Results

The mean age at thelarche in the sample was 12.2 years (median 12, range 4–20 years). Women with thelarche prior to 10 years of age were younger at enrollment and more likely to be non-Hispanic black or Hispanic, be heavier and taller than their peers in childhood, and grow up in a poor family (Table 1). Mean BFHS was similar across age at thelarche groups. Age at thelarche was positively correlated with age at menarche (Spearman *r* = 0.6) and age reached adult height (*r* = 0.3), and negatively correlated with time from thelarche to menarche (*r* = − 0.4) (Additional File 2: Table S1).

**Table 1:** Participant characteristics by age at thelarche among 49,686 women enrolled in the Sister Study cohort

	Age at thelarche							
	< 10 (*N* = 1743)		10–11 (*N* = 13,569)		12–13 (*N* = 25,762)		> 13 (*N* = 8612)	
	Mean	SD	Mean	SD	Mean	SD	Mean	SD
Age at baseline, years	53.9	9.1	55.3	8.9	56.0	8.9	55.3	9.1
Bayesian family history score	0.32	0.09	0.33	0.09	0.33	0.09	0.32	0.09
Age at menarche, years	10.6	1.5	11.6	1.2	12.8	1.1	14.1	1.5
Time from thelarche to menarche, years	1.8	1.6	1.0	1.2	0.4	1.1	−0.6	1.6
Age reached adult height, years	15.0	2.9	15.5	2.5	16.3	2.2	17.2	2.1
Time from menarche to adult height, years	4.4	2.9	3.9	2.5	3.5	2.3	3.1	2.3
	*n*	%	*n*	%	*n*	%	*n*	%
Birth cohort								
1928–1939	138	8	1432	11	3251	13	1047	12
1940–1949	521	30	4472	33	8592	33	2602	30
1950–1959	631	36	5052	37	9606	37	3243	38
1960–1974	453	26	2613	19	4313	17	1720	20
Race/ethnicity								
Non-Hispanic white	1257	72	11,295	83	22,049	86	7050	82
Non-Hispanic black	283	16	1198	9	1952	8	880	10
Hispanic	154	9	735	5	1131	4	413	5
Others	49	3	341	3	630	2	269	3
Family income level growing up								
Well-off	103	6	935	7	1628	6	511	6
Middle income	952	55	8234	61	15,548	60	4940	57
Low income	498	29	3388	25	6639	26	2362	27
Poor	190	11	1012	7	1947	8	799	9
Relative weight to peers at age 10								
Lighter	298	17	3234	24	9197	36	4661	54
Same	700	40	6616	49	12,595	49	3255	38
Heavier	744	43	3693	27	3895	15	674	8
Missing	1		26		75		22	
Relative height to peers at age 10								
Shorter	332	19	2880	21	6612	26	2867	33
Same	649	37	6071	45	12,424	48	3633	42
Taller	760	44	4600	34	6687	26	2107	25
Missing	2		18		39		5	

Column percentages are displayed. Missing data are excluded from percentages. Percentages may not add up to 100 due to rounding

During a mean follow-up of 9.3 years, 3295 of 49,686 eligible women were diagnosed with invasive breast cancer or DCIS (78% and 22% of cases, respectively). Thelarche before 10 years of age was associated with a 23% greater risk of breast cancer compared with thelarche at 12–13 years (95% CI 1.03–1.46; Table 2). Each 1-year delay in age at thelarche was associated with a 3% decrease in breast cancer risk (HR = 0.97, 95% CI 0.95–0.99). Earlier menarche was also associated with an increased risk of breast cancer (HR = 1.10, 95% CI 1.01–1.20 for < 12 years compared with 12–13 years; HR = 0.96, 95% CI 0.94–0.98 for 1-year delay in age at menarche). These associations, particularly age at thelarche, were slightly attenuated when ages at thelarche and menarche were included in the same model.

**Table 2:** Hazard ratios (HRs) and 95% confidence intervals (CIs) for the associations between pubertal timing and tempo and incident breast cancer in the Sister Study cohort

	Person-years	*N* cases	Minimally adjusted^a^		Fully adjusted^b^		Mutually adjusted^c^	
			HR	95% CI	HR	95% CI	HR	95% CI
*Age at pubertal milestone*								
Age at thelarche								
< 10 years	15,806	135	1.22	1.03, 1.46	1.23	1.03, 1.46	1.15	0.95, 1.39
10–11 years	125,803	921	1.02	0.95, 1.11	1.03	0.95, 1.11	0.98	0.90, 1.08
12–13 years	238,712	1725	1.00	Referent	1.00	Referent	1.00	Referent
> 13 years	79,405	514	0.91	0.82, 1.00	0.91	0.82, 1.00	0.93	0.84, 1.04
Continuous (per 1-year later)	459,726	3295	0.97	0.95, 0.99	0.97	0.95, 0.99	0.99	0.96, 1.02
Age at menarche								
< 12 years	93,485	742	1.10	1.01, 1.20	1.10	1.01, 1.20	1.09	0.99, 1.20
12–13 years	258,756	1849	1.00	Referent	1.00	Referent	1.00	Referent
> 13 years	107,484	704	0.92	0.84, 1.00	0.92	0.85, 1.01	0.95	0.86, 1.04
Continuous (per 1-year later)	459,726	3295	0.96	0.94, 0.98	0.96	0.94, 0.98	0.97	0.94, 1.00
Age reached adult height								
< 15 years	116,762	878	0.97	0.89, 1.06	0.97	0.89, 1.06	0.94	0.86, 1.03
15–17 years	188,674	1412	1.00	Referent	1.00	Referent	1.00	Referent
> 17 years	154,289	1005	0.87	0.80, 0.94	0.87	0.80, 0.95	0.88	0.81, 0.95
Continuous (per 1-year later)	459,726	3295	0.99	0.97, 1.00	0.99	0.97, 1.00	0.99	0.98, 1.01
*Pubertal tempo*								
Time from thelarche to menarche								
< 0 years	81,423	592	1.01	0.92, 1.11	1.01	0.92, 1.11	1.06	0.95, 1.17
0 years	169,355	1239	1.00	Referent	1.00	Referent	1.00	Referent
1 year	130,298	913	0.97	0.89, 1.05	0.96	0.89, 1.05	0.95	0.87, 1.03
> 1 year	78,650	551	0.99	0.90, 1.10	1.00	0.90, 1.10	0.96	0.86, 1.06
Continuous (per 1-year longer)	459,726	3295	0.99	0.97, 1.02	0.99	0.97, 1.02	0.97	0.94, 1.00
Time from menarche to adult height								
< 2 years	95,450	702	0.98	0.89, 1.08	0.98	0.89, 1.08	0.99	0.90, 1.09
2–3 years	137,145	973	0.97	0.89, 1.06	0.97	0.89, 1.06	0.98	0.89, 1.07
4–5 years	134,086	971	1.00	Referent	1.00	Referent	1.00	Referent
> 5 years	93,045	649	0.96	0.87, 1.06	0.96	0.87, 1.06	0.93	0.84, 1.03
Continuous (per 1-year longer)	459,726	3295	1.00	0.99, 1.02	1.00	0.99, 1.02	0.99	0.98, 1.01

*N* = 49,686 women included in each model. No violations of proportional hazards assumption for any of the exposures of interest

^a^Adjusted for attained age as the underlying time scale and stratified by birth cohort

^b^Additionally adjusted for race/ethnicity and family income level growing up

^c^Fully adjusted model with mutual adjustment for ages at thelarche and menarche. Age reached adult height is adjusted for age at menarche. Pubertal tempo models are adjusted for age at thelarche (for the thelarche-menarche model) and age at menarche (for the menarche-height model)

**Fig. 1:**
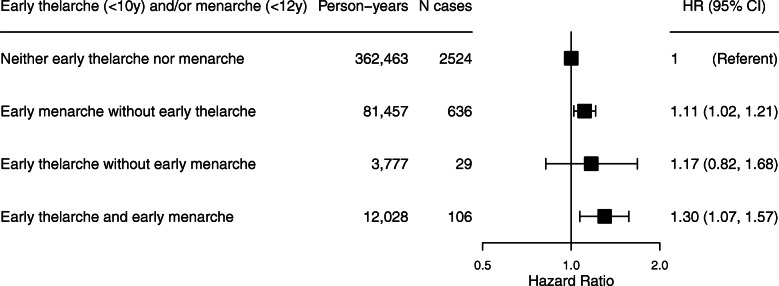
Associations between early thelarche, early menarche, and incident breast cancer in the Sister Study cohort. *N* = 49,686 women. Early thelarche is defined as < 10 years (vs. ≥10 years), and early menarche is defined as < 12 years (vs. ≥12 years). Estimates are adjusted for attained age, race/ethnicity, and family income level growing up and stratified by birth cohort. *p* for interaction between early thelarche and early menarche = 0.99. Hazard ratios (HRs) and 95% confidence intervals (CIs) are plotted on the log scale

Time from thelarche to menarche was not associated with breast cancer risk in models unadjusted for age at thelarche (HR = 0.99, 95% CI 0.97–1.02 per 1-year increase in time from thelarche to menarche), but we observed an inverse association between tempo and risk after adjusting for age at thelarche (HR = 0.97, 95% CI 0.94–1.00; Table 2). The HRs from the categorical model of tempo, although not statistically significant, also suggested an inverse trend after adjustment for age at thelarche. When considering early thelarche (< 10 years) and early menarche (< 12 years) jointly, women with both had a 30% greater risk of breast cancer compared with women with neither risk factor (95% CI 1.07–1.57; Fig. 1).

Reaching adult height at age 18 years or later was associated with a 13% decreased risk of breast cancer compared with reaching adult height at ages 15–17 years (HR = 0.87, 95% CI 0.80–0.95), but there was no increase in risk associated with reaching adult height at an early age (Table 2). These patterns were similar after adjustment for age at menarche, and there was no association between the time from menarche to adult height and breast cancer risk.

**Fig. 2:**
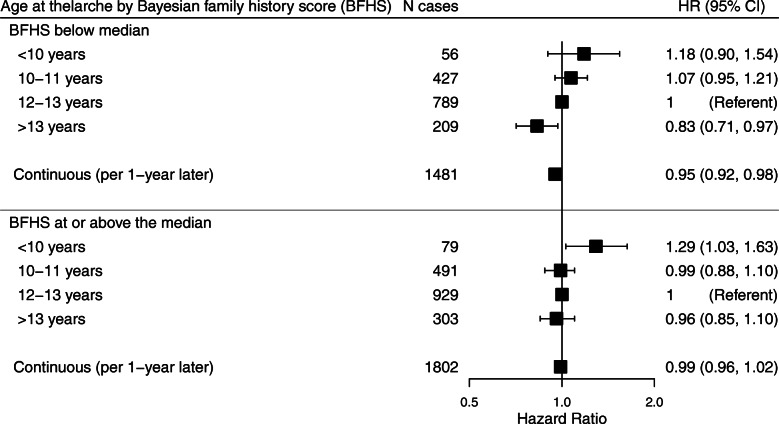
Association between age at thelarche and incident breast cancer stratified by Bayesian family history score. *N* = 49,503 women. Median Bayesian family history score (BFHS) is 0.3076. Estimates are adjusted for attained age, race/ethnicity, and family income level growing up and stratified by birth cohort. *p* for heterogeneity by BFHS is 0.19 for categorical age at thelarche and 0.06 for continuous age at thelarche. Hazard ratios (HRs) and 95% confidence intervals (CIs) are plotted on the log scale

Among women at greater familial risk of breast cancer (BFHS ≥ median), early thelarche (< 10 years) was associated with increased breast cancer risk, while there was no significant decrease in risk associated with late thelarche (Fig. 2). In contrast, age at thelarche had an inverse, linear relationship with breast cancer risk in women with a BFHS below the median, and thelarche after 13 years was associated with a decreased risk. The heterogeneity *p* values for the differences in the association between age at thelarche and breast cancer risk by median BFHS were 0.19 for the categorical exposure model and 0.06 for the continuous model. When BFHS was considered as a continuous variable, the heterogeneity *p* values were 0.33 for the categorical age at thelarche model and 0.86 for the continuous model. The association between age at thelarche and breast cancer risk did not vary by birth cohort, race/ethnicity, or relative weight or height during childhood (Additional File 3: Table S2).

Associations between age at thelarche and breast cancer risk did not differ significantly by tumor invasiveness, menopausal status at diagnosis, or ER status (Additional File 4: Table S3). However, the association between early thelarche and breast cancer risk was only apparent for invasive (HR 1.32, 95% CI 1.08–1.60) and post-menopausal (HR = 1.32, 95% CI 1.09–1.60) disease.

The association between early thelarche and increased breast cancer risk was robust across sensitivity analyses with HRs ranging from 1.18 to 1.31 for < 10 years vs. 12–13 years across models (Additional File 5: Table S4). The results for thelarche-menarche tempo and breast cancer risk were unchanged in sensitivity analyses using a recalculated tempo variable based on adjusting age at thelarche or excluding women ages ≥ 60 years, or in probabilistic bias analyses, with HRs of 0.99–1.00 per 1-year longer tempo.

The inference across all pubertal exposures was similar when we used the same categories for pubertal timing and tempo as in the Generations Study (Additional File 6: Table S5) [10]. Although we did not observe an increased risk associated with early thelarche when defined as ≤ 10 years compared with 11–12 years (HR = 1.02, 95% CI 0.92–1.13), thelarche at ≥ 13 years was associated with decreased breast cancer risk (HR 0.93, 95% CI 0.86–1.00) and breast cancer risk decreased with each category increase in age at thelarche.

## Discussion

Earlier ages at thelarche and menarche were associated with increased breast cancer risk in a prospective cohort of US women with a family history of breast cancer. Having both early thelarche and early menarche was associated with an even greater increase in risk than experiencing only one of these pubertal events at an early age. Reaching adult height at a later age was associated with a modest decrease in breast cancer risk. Neither time from thelarche to menarche nor time from menarche to attainment of adult height was associated with breast cancer risk.

Although breast development begins in utero, the majority occurs after birth and is initiated during puberty [29]. Puberty is triggered by the re-activation of the hypothalamic-pituitary-gonadal axis in childhood. This causes an increase in endogenous hormones; the appearance of physical markers of pubertal development, including breast growth and the pubertal growth spurt in height; and the onset of menses in girls (reviewed in [30]). Hormones and growth factors, particularly estrogen and insulin-like growth factor-1 (IGF-1), work together to regulate breast development during puberty [31, 32]. Rapid breast cell proliferation occurs, including ductal branching and the formation of terminal end buds [29, 32]. These later differentiate into terminal ductal lobular units, the structure within the breast where most cancers originate [29, 33]. However, breast cells do not fully differentiate until the completion of a full-term pregnancy and lactation [8, 31].

Earlier puberty may increase breast cancer risk by increasing the time that breast cells are in a highly proliferative, undifferentiated state that leaves them more susceptible to carcinogenesis [34]. The time between thelarche and menarche is considered biologically relevant to breast cancer risk since the majority of ductal development occurs during this window [10, 29]. Girls who enter puberty earlier experience increases in endogenous hormones at earlier ages. Peak height growth is associated with increased levels of IGF-1, a mitogen that stimulates breast development [35]. Estrogen and progesterone levels rise after menarche, increasing breast cell proliferation [29, 36]. Higher levels of estrogen [37, 38] and IGF-1 [39] in adulthood have been associated with increased breast cancer risk. Earlier and potentially prolonged exposure to high levels during puberty may increase breast cancer risk as well.

We found that earlier age at thelarche, which represents the onset of this period of rapid breast development, was associated with an increased risk of breast cancer. The 23% increased risk associated with thelarche prior to 10 years of age is identical to the association observed in the Generations Study (HR = 1.23, 95% CI 1.02–1.48 for ≤ 10 years vs. 11–12 years) [10], albeit using different cut-offs to define early thelarche. Age at thelarche was inversely associated with the risk of both ER+ and ER− cancers. To our knowledge, the association between age at thelarche and breast cancer risk has not been previously examined by tumor receptor status. The lack of heterogeneity in the age at thelarche association by subtype is consistent with literature suggesting that age at menarche is inversely associated with both hormone receptor-positive and hormone receptor-negative disease [40]. We did not observe significant differences by menopausal status or tumor invasiveness, consistent with the findings in the Generations Study [10]. The positive associations between early thelarche and breast cancer risk in our study were limited to post-menopausal breast cancer and invasive disease, though there were relatively few pre-menopausal or DCIS cases.

Age at thelarche has declined over time [2], and this secular trend is evident in our cohort as women born after 1960 were more likely to report earlier thelarche. The inverse association between age at thelarche and breast cancer risk was observed across the birth cohorts in our study, spanning from the 1930s to the 1970s. Data from the USA and Europe suggest that age at thelarche has continued to decline, with a mean age of less than 10 years observed in recent pubertal cohorts [4, 5]. Given this continued secular decline, early age at thelarche may contribute to future trends in breast cancer incidence.

The attributable risk of breast cancer due to earlier puberty may be higher in populations where early thelarche is more common. Age at thelarche is earlier, on average, in black and Hispanic girls in the USA than in non-Hispanic white girls [4, 19]. In our cohort, non-Hispanic black and Hispanic women were more likely to report early thelarche than non-Hispanic white women. Thus, while the association between age at thelarche and breast cancer risk did not vary meaningfully by race/ethnicity, it may still contribute differentially to the burden of breast cancer among women of different racial/ethnic groups. Girls that are overweight in childhood are more likely to experience earlier thelarche [20], but childhood adiposity is associated with decreased breast cancer risk [41, 42]. Our finding that the association between age at thelarche and breast cancer risk was not modified by relative childhood weight suggests that early puberty and childhood weight affect risk through different pathways and that early thelarche should be considered a risk factor in both overweight and non-overweight girls. The relationship between earlier thelarche and increased breast cancer risk was observed in women that were taller than their peers in childhood, a group at increased risk of both early puberty [43] and breast cancer [44], as well as in women that were the same height or shorter than their peers.

Women in our study have at least one sister with breast cancer and have, on average, approximately twice the risk of breast cancer than women without a first-degree family history [45]. The consistency of the age at thelarche association in our cohort and the Generations Study, in which only 15% of the study population had a first-degree family history [10], suggests that early thelarche is a risk factor for breast cancer in women with and without a family history. Studies of twin pairs have suggested that earlier thelarche may be associated with increased breast cancer risk and earlier age at onset among women with a family history of breast cancer [46, 47]. In our study, early thelarche (< 10 years) was associated with increased breast cancer risk across the range of first-degree family history captured through the BFHS, although the association was statistically significant and the point estimate higher for women with a BFHS at or above the median. For late age at thelarche (> 13 years), hazard ratios in both groups suggested an association with decreased breast cancer risk, but the estimate for women with a BFHS below the median was further from the null and statistically significant. While this apparent difference in results is intriguing, there is no evidence that the findings differ significantly by family history score, so we are reluctant to speculate about possible biological explanations. Furthermore, the mean BFHS did not vary by age at thelarche. However, since women with a family history have a greater underlying risk of breast cancer, the increased risk associated with early thelarche will be larger on an absolute scale in these women compared with women who are not at increased familial risk [48].

Consistent with prior studies [1], we also observed an inverse association between age at menarche and breast cancer risk. It is difficult to disentangle the influence of early thelarche from early menarche, given the high correlation between ages at these events. However, we observed a greater increased risk of breast cancer associated with experiencing both milestones at an early age than experiencing early thelarche or early menarche only, suggesting that the timing of both markers contributes to risk. While age at menarche is often collected in research settings, age at thelarche is not commonly assessed. More studies should collect data on age at thelarche to further explore the independent contribution of age at thelarche to breast cancer risk and examine the utility of using this information to improve risk assessment. In the future, routine collection of age at thelarche in clinical settings and inclusion in risk assessment models may help to identify high-risk women that may benefit from increased screening or risk reduction measures.

We did not observe an association between the time from thelarche to menarche, or tempo, and breast cancer risk, in contrast to the positive association observed in the Generations Study [10]. Our cohort was older on average than the Generations cohort at enrollment, but the inference was unchanged when we limited the tempo analysis to women less than 60 years at baseline. Misreporting of pubertal timing, particularly age at thelarche, may have limited our ability to detect an association between thelarche-menarche tempo and breast cancer risk. Age at menarche can be reliably recalled in adulthood [49], but the reliability and validity of recalled age at thelarche is not known. The mean age at menarche in the cohort was 12.6 years, which is similar to data from NHANES [50]. The mean age at thelarche was 12.2 years and the mean thelarche-menarche tempo was 0.4 years in our cohort. In comparison, longitudinal studies of women born in the same birth cohorts as our study population observed that thelarche occurred on average around 11 years of age and approximately 2 years prior to menarche (reviewed in [3]). This suggests that women tended to recall a later age at thelarche than when it occurred, leading to an underestimation of pubertal tempo and a potential bias towards the null in our analysis of tempo and breast cancer risk. In our cohort, 37% of women reported that thelarche occurred at the same age as menarche, which likely reflects misreporting of age at thelarche as well as reduced precision of the tempo assessment since thelarche and menarche were reported to the nearest year. Since women are regularly asked about age at menarche but not thelarche, it is possible that women who were reluctant to answer “do not know” assumed thelarche must have been at around the same time as menarche. In sensitivity analyses, we considered whether inaccuracies in reporting age at thelarche explained the lack of association between tempo and breast cancer risk but results were unchanged. However, tempo was positively associated with breast density in a subset of the Generations Study [51] and in the prospective Dietary Intervention Study in Children follow-up study [52]. We therefore would not rule out an association between tempo and breast cancer risk but suggest that additional studies with prospective assessments of thelarche and menarche will be necessary to limit measurement error in estimates of the association between tempo and breast cancer risk.

We also observed a decreased risk of breast cancer associated with reaching adult height at a later age. Our findings are consistent with previous studies [10, 13–17], though no association was observed in pre-menopausal women in the Nurses’ Health Study II cohort [18]. We did not observe an association between time from menarche to adult height and breast cancer risk, similar to others [10, 18]. Earlier age reaching adult height is correlated with an earlier age at peak height velocity and a faster rate of height growth [11], which have both been associated with increased breast cancer risk [44].

Strengths of this study include the large sample size; prospective design, which limited differential recall of pubertal timing by breast cancer status; and inclusion of multiple pubertal markers. We were able to assess whether the association between age at thelarche and breast cancer risk varied by breast cancer characteristics including ER status. We further examined effect modification by factors associated with early thelarche, including race/ethnicity, childhood body size, and extent of breast cancer family history, which to our knowledge have not been investigated previously.

Analyses were limited by recalled data on ages at pubertal milestones, which may be reported with error. We categorized the exposures to limit bias resulting from measurement error, although we also examined ages at pubertal milestones continuously to facilitate comparisons with prior studies. The validity of age at menarche, for example, has been shown to be improved when categorized as early, normal, and late [53]. Age at thelarche in particular may be difficult for women to accurately recall in adulthood. Sensitivity analyses to assess the influence of misclassification of age at thelarche suggest that inaccurate recall does not account for the association observed between earlier thelarche and increased breast cancer risk, as we observed minimal attenuation of the hazard ratios in quantitative bias analyses. However, inaccuracies in self-reports of age at thelarche may have limited our ability to detect an association between thelarche-menarche tempo and breast cancer risk. As a derived variable, tempo may be more sensitive to reporting errors in the exact ages of thelarche and menarche than analyses examining the timing of these milestones individually. In addition, multiple testing of correlated pubertal exposures increased the likelihood of a false-positive finding. We also had reduced precision in stratified analyses for small subgroups, such as some racial/ethnic groups. We did not have the temporal data necessary to examine whether early-life body size confounded or mediated the relationship between age at thelarche and breast cancer risk since body size was reported for age 10 and age at thelarche ranged from 4 to 20 years. Although all women in our cohort have a sister with breast cancer, the consistency of our findings with other studies suggests that our results may still be generalizable to women without a family history.

## Conclusions

Our findings suggest that earlier age at attainment of pubertal milestones may enhance susceptibility to breast carcinogenesis. Early age at thelarche is an emerging breast cancer risk factor that may have considerable public health impact, given secular declines in age at thelarche in the USA and abroad. Studies with prospective measures of pubertal tempo are needed to clarify whether the time between thelarche and menarche is a particularly vulnerable period in relation to breast cancer risk.

## Supplementary information

**Additional file 1: Figure S1.** Flow chart of eligible study population.

**Additional file 2: Table S1.** Spearman correlations between pubertal variables

**Additional file 3: Table S2.** Hazard ratios (HRs) and 95% confidence intervals (CIs) for the association between age at thelarche and incident breast cancer in the Sister Study cohort stratified by characteristics associated with age at thelarche

**Additional file 4: Table S3.** Hazard ratios (HRs) and 95% confidence intervals (CIs) for the association between age at thelarche and incident breast cancer in the Sister Study cohort by breast cancer characteristics

**Additional file 5: Table S4.** Sensitivity analyses for the association between age at thelarche and incident breast cancer in the Sister Study cohort

**Additional file 6: Table S5.** Hazard ratios (HRs) and 95% confidence intervals (CIs) for the associations between pubertal timing and tempo and incident breast cancer in the Sister Study cohort using the same categories as in the Generations Study [10]

## Data Availability

Requests for data, including the data used in this manuscript, are welcome. De-identified data is made available upon request as described on the study website (https://sisterstudy.niehs.nih.gov/English/data-requests.htm). The data sharing policy was developed to protect the privacy of study participants and is consistent with study informed consent documents as approved by the NIEHS Institutional Review Board.

## Abbreviations

BFHS: Bayesian family history score
CI: Confidence interval
DCIS: Ductal carcinoma in situ
ER: Estrogen receptor
HR: Hazard ratio
IGF-1: Insulin-like growth factor-1
