# Supplementary figures and images for: Pubertal timing and breast cancer risk in the Sister Study cohort
Source: Breast Cancer Res. 2020 Oct 27;22:112. doi: 10.1186/s13058-020-01326-2 (PMC7590599; doi:10.1186/s13058-020-01326-2)

**Figure S1.** Flow chart of eligible study population.

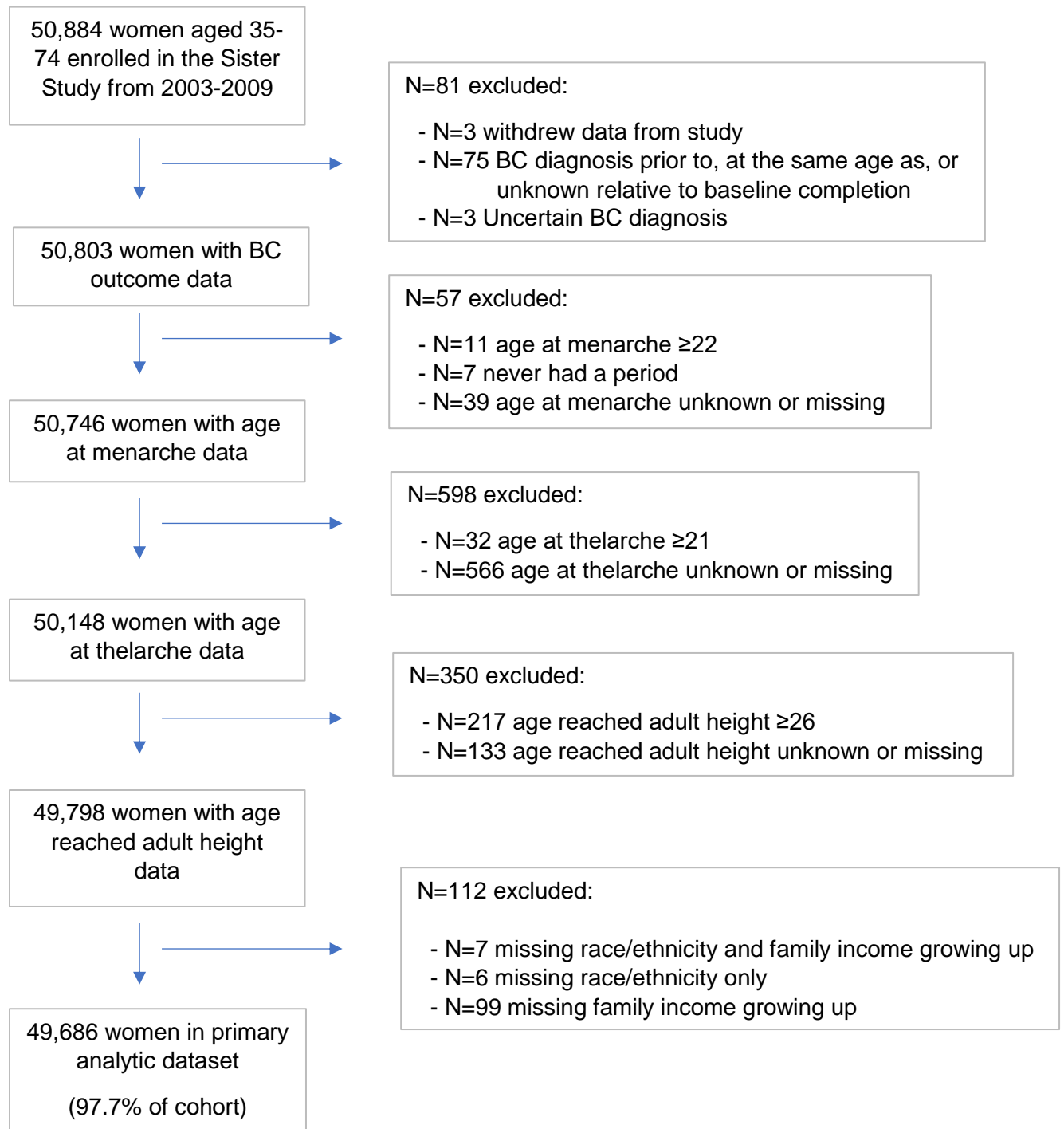

Supplement: Supplementary file 1 — Additional file 1: Figure S1. Flow chart of eligible study population. [file 13058_2020_1326_MOESM1_ESM.pdf]
