# Supplementary material for: Pubertal timing and breast cancer risk in the Sister Study cohort
Source: Breast Cancer Res. 2020 Oct 27;22:112. doi: 10.1186/s13058-020-01326-2 (PMC7590599; doi:10.1186/s13058-020-01326-2)
Supplement: Supplementary file 2 — Additional file 2: Table S1. Spearman correlations between pubertal variables [file 13058_2020_1326_MOESM2_ESM.pdf]

Table S1. Spearman correlations between pubertal variables

	Age at thelarche	Age at menarche	Age reached adult height	Time from thelarche to menarche
Age at menarche	0.62			
Age reached adult height	0.26	0.28		
Time from thelarche to menarche	-0.44	0.36	0.01	
Time from menarche to adult height	-0.11	-0.31	0.79	-0.21

All correlations coefficients significant at p<.0001 except correlation between age reached adult height and time between thelarche and menarche
