# Supplementary material for: Pubertal timing and breast cancer risk in the Sister Study cohort
Source: Breast Cancer Res. 2020 Oct 27;22:112. doi: 10.1186/s13058-020-01326-2 (PMC7590599; doi:10.1186/s13058-020-01326-2)
Supplement: Supplementary file 3 — Additional file 3: Table S2. Hazard ratios (HRs) and 95% confidence intervals (CIs) for the association between age at thelarche and incident breast cancer in the Sister Study cohort stratified by characteristics associated with age at thelarche [file 13058_2020_1326_MOESM3_ESM.pdf]

Table S2. Hazard ratios (HRs) and 95% confidence intervals (CIs) for the association between age at thelarche and incident breast cancer in the Sister Study cohort stratified by characteristics associated with age at thelarche

	Age at thelarche													Continuous		
	<10 years			10-11 years			12-13 years			>13 years			P-het[a]	Per 1-year later		P-het[b]
	N cases	HR	95% CI	N cases	HR	95% CI	N cases	HR	95% CI	N cases	HR	95% CI		HR	95% CI	
Birth cohort[c]													0.64			0.52
1928-1939	16	1.42	0.85, 2.35	109	0.87	0.70, 1.09	282	1	Referent	79	0.85	0.66, 1.10		0.99	0.93, 1.05	
1940-1949	41	1.04	0.76, 1.43	364	1.09	0.96, 1.24	641	1	Referent	180	0.92	0.78, 1.09		0.98	0.94, 1.01	
1950-1959	50	1.42	1.06, 1.89	315	1.07	0.93, 1.23	564	1	Referent	171	0.91	0.77, 1.09		0.95	0.91, 0.98	
1960-1974	28	1.19	0.80, 1.76	133	0.93	0.75, 1.14	238	1	Referent	84	0.88	0.69, 1.14		0.98	0.93, 1.04	
Race/ethnicity[d]													0.64			0.63
Non-Hispanic white	105	1.26	1.03, 1.53	772	1.00	0.92, 1.09	1517	1	Referent	438	0.91	0.82, 1.01		0.98	0.95, 1.00	
Non-Hispanic black	16	1.04	0.62, 1.76	78	1.17	0.87, 1.57	111	1	Referent	45	0.94	0.67, 1.34		0.95	0.89, 1.02	
Hispanic	10	1.46	0.74, 2.88	38	1.11	0.72, 1.69	54	1	Referent	19	0.93	0.55, 1.58		0.97	0.87, 1.09	
Other	4	1.16	0.41, 3.27	33	1.39	0.88, 2.21	43	1	Referent	12	0.63	0.33, 1.21		0.91	0.80, 1.03	
Weight relative to peers at age 10[e]													0.79			0.82
Same weight or lighter	81	1.26	1.01, 1.58	712	1.05	0.96, 1.15	1521	1	Referent	478	0.88	0.79, 0.98		0.96	0.94, 0.98	
Heavier	54	1.43	1.05, 1.93	208	1.09	0.89, 1.32	201	1	Referent	36	1.01	0.70, 1.45		0.95	0.90, 1.01	
Height relative to peers at age 10[e]													0.62			0.57
Same height or shorter	74	1.27	1.00, 1.61	588	1.05	0.95, 1.15	1213	1	Referent	380	0.93	0.83, 1.04		0.97	0.94, 1.00	
Taller	61	1.10	0.84, 1.44	333	0.95	0.83, 1.09	510	1	Referent	134	0.85	0.70, 1.03		0.98	0.95, 1.02	

No violations of proportional hazards assumption for any of the exposures of interest.
[a]P for heterogeneity from joint Wald test using fully adjusted Cox model
[b]P for heterogeneity from Wald or joint Wald test using fully adjusted Cox model
[c]Adjusted for attained age, race/ethnicity and family income level growing up
[d]Adjusted for attained age and family income level growing up and stratified by birth cohort
[e]Adjusted for attained age, race/ethnicity and family income level growing up and stratified by birth cohort
