# Supplementary material for: Pubertal timing and breast cancer risk in the Sister Study cohort
Source: Breast Cancer Res. 2020 Oct 27;22:112. doi: 10.1186/s13058-020-01326-2 (PMC7590599; doi:10.1186/s13058-020-01326-2)
Supplement: Supplementary file 4 — Additional file 4: Table S3. Hazard ratios (HRs) and 95% confidence intervals (CIs) for the association between age at thelarche and incident breast cancer in the Sister Study cohort by breast cancer characteristics [file 13058_2020_1326_MOESM4_ESM.pdf]

Table S3. Hazard ratios (HRs) and 95% confidence intervals (CIs) for the association between age at thelarche and incident breast cancer in the Sister Study cohort by breast cancer characteristics

	Age at thelarche													Continuous		
	<10 years			10-11 years			12-13 years			>13 years			P-het	Per 1-year later		P-het
	N cases	HR	95% CI	N cases	HR	95% CI	N cases	HR	95% CI	N cases	HR	95% CI		HR	95% CI	
Tumor invasiveness[a,b]													0.52			0.26
Invasive	112	1.32	1.08, 1.60	717	1.03	0.94, 1.12	1345	1	Referent	398	0.90	0.80, 1.01		0.96	0.94, 0.99	
DCIS	23	0.93	0.61, 1.42	204	1.02	0.86, 1.21	380	1	Referent	116	0.92	0.75, 1.14		0.99	0.95, 1.04	
Estrogen receptor (ER) status[a,b]													0.95			0.55
ER+	93	1.15	0.94, 1.43	667	0.99	0.90, 1.09	1299	1	Referent	365	0.86	0.77, 0.97		0.97	0.95, 1.00	
ER-	20	1.32	0.83, 2.08	117	0.96	0.77, 1.20	233	1	Referent	66	0.85	0.65, 1.12		0.95	0.90, 1.01	
Menopausal status[c,d]													0.29			0.10
Pre-menopausal	24	0.94	0.62, 1.42	156	0.91	0.75, 1.11	308	1	Referent	98	0.83	0.66, 1.04		1.00	0.96, 1.05	
Post-menopausal[e]	111	1.32	1.09, 1.60	765	1.06	0.97, 1.15	1417	1	Referent	416	0.92	0.83, 1.03		0.96	0.94, 0.98	

[a]Joint Cox model adjusted for attained age, race/ethnicity and family income level growing up and stratified by birth cohort and subtype

[b]P for heterogeneity from joint Wald test for categorical exposure or Wald test for continuous exposure from a fully adjusted joint Cox proportional hazards model

[c]Adjusted for attained age, race/ethnicity and family income level growing up and stratified by birth cohort

[d]P for heterogeneity from joint Wald test for categorical exposure or Wald test for continuous exposure using fully adjusted Cox model

[e]Proportional hazards assumption violated for continuous age at thelarche exposure in strata of post-menopausal women
