# Supplementary material for: Pubertal timing and breast cancer risk in the Sister Study cohort
Source: Breast Cancer Res. 2020 Oct 27;22:112. doi: 10.1186/s13058-020-01326-2 (PMC7590599; doi:10.1186/s13058-020-01326-2)
Supplement: Supplementary file 5 — Additional file 5: Table S4. Sensitivity analyses for the association between age at thelarche and incident breast cancer in the Sister Study cohort [file 13058_2020_1326_MOESM5_ESM.pdf]

Table S4. Sensitivity analyses for the association between age at thelarche and incident breast cancer in the Sister Study cohort

			Age at thelarche[a]												Continuous (per 1-year later)	
			<10 years			10-11 years			12-13 years			>13 years				
Analysis	N in model	Person-years	N cases	HR	95% CI	N cases	HR	95% CI	N cases	HR	95% CI	N cases	HR	95% CI	HR	95% CI
Full cohort	49,686	459,726	135	1.23	1.03, 1.46	921	1.03	0.95, 1.11	1725	1	Referent	514	0.91	0.82, 1.00	0.97	0.95, 0.99
Adjusting age at thelarche:																
Assuming age at thelarche occurred one year earlier than reported for women with thelarche-menarche tempo ≤0 years	49,686	459,726	227	1.18	1.02, 1.36	1388	1.11	1.03, 1.20	1380	1	Referent	300	1.00	0.88, 1.13	0.96	0.94, 0.99
Assuming age at thelarche occurred two years earlier than reported for women with thelarche-menarche tempo ≤0 years	49,686	459,726	482	1.19	1.07, 1.33	1626	1.07	0.99, 1.16	997	1	Referent	190	0.94	0.80, 1.09	0.96	0.94, 0.99
Restricting study population:																
Excluding women age ≥60 years at baseline	33,506	314,210	93	1.30	1.05, 1.61	581	1.05	0.95, 1.16	1028	1	Referent	324	0.93	0.82, 1.06	0.96	0.94, 0.99
Excluding women with thelarche-menarche tempo below 5th percentile (-2 years) or above the 95th percentile (3 years)	47,762	442,344	116	1.21	1.00, 1.46	900	1.02	0.94, 1.11	1705	1	Referent	464	0.90	0.81, 1.00	0.97	0.95, 0.99
Excluding women with thelarche-menarche tempo <0 years or >4 years	40,547	375,929	127	1.23	1.03, 1.48	849	1.02	0.94, 1.11	1437	1	Referent	271	0.88	0.77, 1.00	0.97	0.94, 1.00

Excluding women with thelarche-menarche tempo ≤0 years (thelarche at the same age or after menarche)	22,476	208,948	109	1.31	1.07, 1.60	574	1.03	0.92, 1.15	673	1	Referent	108	0.95	0.77, 1.16	0.97	0.93, 1.01

No violations of proportional hazards assumption for any of the exposures of interest.

[a]Adjusted for attained age as the underlying time scale, race/ethnicity and family income level growing up and stratified by birth cohort
