# Supplementary material for: Pubertal timing and breast cancer risk in the Sister Study cohort
Source: Breast Cancer Res. 2020 Oct 27;22:112. doi: 10.1186/s13058-020-01326-2 (PMC7590599; doi:10.1186/s13058-020-01326-2)
Supplement: Supplementary file 6 — Additional file 6: Table S5. Hazard ratios (HRs) and 95% confidence intervals (CIs) for the associations between pubertal timing and tempo and incident breast cancer in the Sister Study cohort using the same categories as in the Generations Study [10] [file 13058_2020_1326_MOESM6_ESM.pdf]

Table S5. Hazard ratios (HRs) and 95% confidence intervals (CIs) for the associations between pubertal timing and tempo and incident breast cancer in the Sister Study cohort using the same categories as in the Generations Study (Bodicoat et al, 2014)

	Person-years	N cases	Minimally adjusted[a]		Fully adjusted[b]	
			HR	95% CI	HR	95% CI
Age at pubertal milestone						
Age at thelarche						
≤10 years	61,777	454	1.02	0.92, 1.13	1.02	0.92, 1.13
11-12 years	220,129	1623	1.00	Referent	1.00	Referent
≥13 years	177,819	1218	0.93	0.86, 1.00	0.93	0.86, 1.00
HR for trend[c]	459,726	3295	0.95	0.90, 1.00	0.95	0.90, 1.00
Age at menarche						
≤12 years	218,141	1639	1.07	1.00, 1.15	1.08	1.00, 1.16
13-14 years	196,181	1369	1.00	Referent	1.00	Referent
≥15 years	45,403	287	0.91	0.81, 1.04	0.92	0.81, 1.04
HR for trend[c]	459,726	3295	0.93	0.88, 0.98	0.93	0.88, 0.98
Age reached adult height						
≤14 years	116,762	878	0.97	0.89, 1.06	0.97	0.89, 1.06
15-16 years	133,583	1006	1.00	Referent	1.00	Referent
≥17 years	209,381	1411	0.91	0.84, 0.98	0.91	0.84, 0.98
HR for trend[c]	459,726	3295	0.96	0.92, 1.00	0.96	0.92, 1.00
Pubertal tempo						
Time from thelarche to menarche						
<0 years	81,423	592	1.01	0.92, 1.11	1.01	0.92, 1.11
0 years	169,355	1239	1.00	Referent	1.00	Referent
1 year	130,298	913	0.97	0.89, 1.05	0.96	0.89, 1.05
≥2 years	78,650	551	0.99	0.90, 1.10	1.00	0.90, 1.10
HR for trend[c]	459,726	3295	0.99	0.96, 1.03	0.99	0.96, 1.03
Time from menarche to adult height						
<0 years	10,239	76	1.01	0.79, 1.28	1.01	0.79, 1.28
0-1 years	85,211	626	1.00	Referent	1.00	Referent
2-3 years	137,145	973	0.99	0.90, 1.10	0.99	0.90, 1.10
≥4 years	227,131	1620	1.01	0.92, 1.11	1.01	0.92, 1.11
HR for trend[c]	459,726	3295	1.00	0.96, 1.05	1.01	0.97, 1.05

N=49,686 women included in each model. No violations of proportional hazards assumption for any of the exposures of interest.

[a]Adjusted for attained age and stratified by birth cohort
[b]Additionally adjusted for race/ethnicity and family income level growing up
[c]HR for trend corresponds to a one-category increase in exposure, modelled as an ordinal variable
